# Concomitant ablation for atrial fibrillation during septal myectomy in patients with hypertrophic obstructive cardiomyopathy

**DOI:** 10.1186/1749-8090-10-S1-A139

**Published:** 2015-12-16

**Authors:** A Bogachev-Prokophiev, S Zheleznev, M Fomenko, A Pivkin, A Afanasiev, R Sharifulin, A Karaskov

**Affiliations:** 1 Heart Valves Surgery Department, State Research Institute of Circulation Pathology, Novosibirsk, Russian Federation

## Background/Introduction

Atrial fibrillation (AF) is the most common tachyarrhythmia in patients with hypertrophic obstructive cardiomyopathy (HOCM). AF emergence associated with significant clinical deterioration in patients with HOCM, that's why maintenance of sinus rhythm is desirable. The most papers are reported results about catheter AF ablation but only paper including date about concomitant AF ablation during septal myectomy.

## Aims/Objectives

Aim of the study was evaluation efficacy of concomitant AF ablation during septal myectomy in patients with HOMC.

## Method

Between 2010 and 2013, 187 patients underwent of extended myectomy procedures. In 45 cases was performed concomitant AF ablation. AF was paroxysmal in 26 (58%), persistent in 19 (42%). Mean age was 52.8 ± 14.2 years (range 22 to 74 years). A primary HOCM was the main indication for surgery according to 2011 ACCF/AHA guidelines. Mean peak gradient was 90.7 ± 24.2 mm Hg, thickness of interventricular septum was 26.1 ± 4.3 mm. Mean AF duration was 17 ± 8 months.

## Results

There were no early death. No procedure-related complications occurred with regard to ablation procedure. Complete atrioventricular block was in 2 (4.0%) cases with dual-chamber pacemaker implantation. Mean time cross clamping was 61.7 ± 26.2 min. Peak LVOT gradient was 14.6 ± 5.5 mmHg. Ablation technique was maze IV procedure for all patients (RF ablation with bipolar clamp + cryo lesion for mitral and tricuspid lines). Because of atrial wall thickness (5-6 mm) applications performed 8-10 times at the same line. There were no pacemaker implantation due to sinus node dysfunction. All patients were discharged in stable sinus rhythm. Mean follow-up was 24 ± 7 months. AF freedom at 6 months was 100% (45 pts), at 1 year was 93.3% (42 pts) and at 21 months was 82.2% (37 pts).

## Discussion/Conclusion

Concomitant ablation for AF during septal myectomy in patients with HOCM safe and effective procedure, and should be considered carefully in these kind patients.

